# Associations between number of pain sites and sleep, sports participation, and quality of life: a cross-sectional survey of 1021 youth from the Midwestern United States

**DOI:** 10.1186/s12887-019-1576-6

**Published:** 2019-06-17

**Authors:** David M. Bazett-Jones, Michael S. Rathleff, Sinead Holden

**Affiliations:** 1 Department of Athletic Training, University of Toledo, Health & Human Services, Mail Stop 119, 2801 W. Bancroft St, Toledo, OH 43606-3390 USA; 2 Research Unit for General Practice in Aalborg, Department of Clinical Medicine, Aalborg University, Aalborg, Denmark; 3 SMI, Department of Health Science and Technology, Aalborg University, Aalborg, Denmark

**Keywords:** EQ-5D, Adolescents, Children

## Abstract

**Background:**

Musculoskeletal pain in youth is common but little is known about the influence of the number of pain sites on pain characteristics. The objective of this study was to compare pain characteristics, quality of life, sleep, sport participation between adolescents without pain, those with single site pain, and those with multi-site pain and investigate the relationship between pain duration and number of pain sites.

**Methods:**

An online survey was sent via email to 7177 possible middle- and high-school students. The students completed a survey containing questions about their pain (including location, duration, intensity, frequency), health-related quality of life, sleep quantity and quality, and sports participation. Quantitative variables were analysed with one-way ANOVAs or t-tests and qualitative variables were analysed with Pearson Chi-squared tests. Relationships were investigated with a Pearson Correlation.

**Results:**

Of the respondents (*n* = 1021), 52.9% reported no pain, 17.2% reported pain in a single-site, and 29.9% reported pain in multiple sites. Those with multi-site pain reported significantly lower quality of life than both pain-free youth (*p* < 0.001) and those with single-site pain (*p* < 0.001); those with single-site pain had lower quality of life than pain-free youth (*p* < 0.001). Those with pain reported worse sleep than those without pain (*P* < 0.05). No differences in sport participation were found (*p* > 0.10). Those with multi-site pain reported greater intensity (*p* = 0.005) and duration (*p* < 0.001) than those with single-site pain. A positive, moderate, and significant correlation (*r* = 0.437, p < 0.001) was found between the pain duration and number of pain sites.

**Conclusions:**

A large percentage of youth experience regular pain that affects their self-reported quality of life and sleep, with greater effects in those with multi-site pain.

## Background

Musculoskeletal (MSK) pain is a common complaint in adolescents. Approximately one third of adolescents experience regular (at least monthly) MSK pain [1]. Although the estimates vary between studies and populations, sports active adolescents appear to have the highest prevalence of pain complaints [2]. A concern is that adolescent pain complaints are not always transient, and 50% will continue to experience pain even years later [3]. Adolescent MSK pain is a public health concern, as it is the largest contributor to years lived with disability, which increases rapidly during the transition from childhood into adolescence [4]. The presence of persistent pain during adolescence can have a negative effect on physical activity, health-related quality of life (HRQoL), anxiety, school attendance, participation in hobbies and social activities, and can cause disturbances in appetite, sleep and mental health [5–7].

The presentation of MSK pain complaints in adolescents vary considerably, ranging from localised pain with a short duration [8], all the way to wide-spread multi-site pain [9], and can last through to adulthood [10]. Multi-site pain is common, with one in three of all adolescents (12–19 years) reporting pain in more than one location [11]. A greater number of pain sites appear to have a larger impact on physical and social activity, relative to localised pain [12]. Similarly, data have shown a link between multi-site pain during adolescence and future mental health disorders [13]. Despite the commonality of multi-site pain, the majority of previous research has focused on specific pain locations, such as the knee or back [14–16], neglecting the presence of co-occurring pain sites. However, the number of pain sites, may be of particular importance to investigate, as pain in multiple locations may indicate a progression of long-standing pain [17] and be indicative of a poorer prognosis [18].

Furthermore, adolescents’ pain experience may be modulated by lifestyle factors (sports participation, sleep) and psychosocial factors (quality of life). These factors represent potentially modifiable factors, and the question remains whether these factors are differentially associated with the number of pain sites or the location of MSK pain. Understanding their association to the adolescent’s pain experience may present a way to gain insight into the determinants of their pain experience and inform suitable treatment target.

## Methods

This paper aims to 1) compare pain characteristics (intensity, duration), quality of life, sleep, sport participation between adolescents without pain, those with single site pain, and those with multi-site pain and 2) investigate the relationship between pain duration and number of pain sites.

### Study design and recruitment

This cross-sectional survey study was conducted in January of 2017. The reporting of the study follow the STROBE guidelines [19]. This survey was conducted in a suburban school district in the Midwestern United States. The school district approved this study, as did the University’s Institutional Review Board. All middle- and high-school students (ages 10–18) and parents were sent information about this study and were given the opportunity to request that they be removed from the recruitment email list. Per parental request, a total of 33 potential participants were removed from the survey pool. A link to the final survey was sent to 7177 total students. Participants were able to respond to the link and indicate that they were not interested in participating in the study. Regardless of participation, each respondent was entered into a drawing for a $50 gift card.

### Survey

The survey was designed to explore pain characteristics, HRQoL, sleep and sports participation. We used specific questions within these four domains, drawn from previous (population based) studies conducted in adolescent populations [20–22]. The survey was constructed in Google forms.

### Pain

Participants were asked to indicate if they had experienced pain in the previous six months in any of nine predefined locations, and if they were currently experiencing pain in these locations. If current pain was reported, then additional variables of location, age of onset (i.e. duration) and, frequency of pain, average and worst pain (measured on a numerical rating scale from 0 to 10, with 0 being no pain in the last week and 10 being the worst pain imaginable). Participants were asked to report their average and worst pain over the last week. Participants were then asked about the location of pain with the options being neck, upper back, lower back, hips, knees, ankles/feet, shoulders, elbows, and wrists/hands. Participants were asked to report age when the pain first started, which was used to calculate pain duration (in years). The frequency of pain was assessed using the following options “Almost Daily”, “Several times a week”, “Weekly”, “Monthly”, and “Rarely”.

### Health related quality of life

Health-related quality of life was assessed with the EuroQol Group 5-Dimensional 3 levels Self-Report Questionnaire (EQ-5D-3 L). The EQ-5D is a general health questionnaire where participants report the problems (none, some or a lot) in the areas of walking about, washing/dressing, doing usual activates, pain/discomfort, and feelings of worry, sadness, or unhappiness. To calculate the index score we used the time trade-off method specific for the US population [23]. The index score for EQ-5D ranges from − 0.59 to 1.00, with higher scores indicate better quality of life.

### Sleep

Sleep quality was assessed using methods described by Auvinen et al. [21]. Participants were asked “How well does each statement apply at present, or over the past 6 months?” (1) “I have nightmares”, (2) “I am too tired” and (3) “I have sleep problems”. Participants could respond to these statements as “Never”, “To some extent or sometimes”, and “Very much or often”. Sleep quantity (average hours/night) was assessed via participants self-report [22]. Based on these qualitative and quantitative responses, all participants were categorized as having (1) sufficient sleep (8–9 h per day and no nightmares, tiredness or general sleep problems), (2) intermediate sleep (7 or 10 or more h per day, or having nightmares, tiredness or general sleep problems to some extent or sometimes) or (3) insufficient sleep (6 h or less per day, or often having nightmares, tiredness, or general sleep problems) [21].

### Sports participation

Participants were asked if they participated in sports (yes/no), and if yes, the number of days and hours of sport participation per week. Weekly sports participation was computed by multiplying the number of days per week of sports participation by the average daily hours of sports participation [22]. Participants self-reported sex, age, height, and mass. Height and mass were used to calculate BMI (kg/m^2^).

### Data analyses

For this study, participants were included in the single site-pain group if they described current pain in one site, irrespective of location on the body. If they reported pain in more than one location, they were included in the multi-site pain group. The no-pain (control) group was defined as youth reporting no pain in the previous six months. Those who reported no current pain, but pain in the last six months were excluded from the analysis (*n* = 106).

To compare quantitative variables (age, height, weight, BMI, EQ-5D index scores, sleep hours, sports participation hours and HRQoL) among groups (single-site versus multi-site versus control), one-way analyses of variance (ANOVA) were utilized. Pairwise comparisons were completed using Tukey tests for significant main effects. Pearson chi-squared tests were used for categorical and dichotomous variables (sex, pain frequency, sleep quality, sports participation). Pairwise comparisons were performed using chi-squared tests with Bonferroni corrections. Comparisons of pain intensity (average, worst) and duration were performed with independent t-tests. To investigate the relationship between pain duration (pain site with longest duration) and the total number of pain sites, a Pearson Correlation was used. Pearson correlation magnitudes were interpreted as small (0–0.3), moderate (0.3–0.5), large (0.5–0.7), and very large (0.7–1.0) [24]. Significance was set at *p* < 0.05.

## Results

**Fig. 1:**
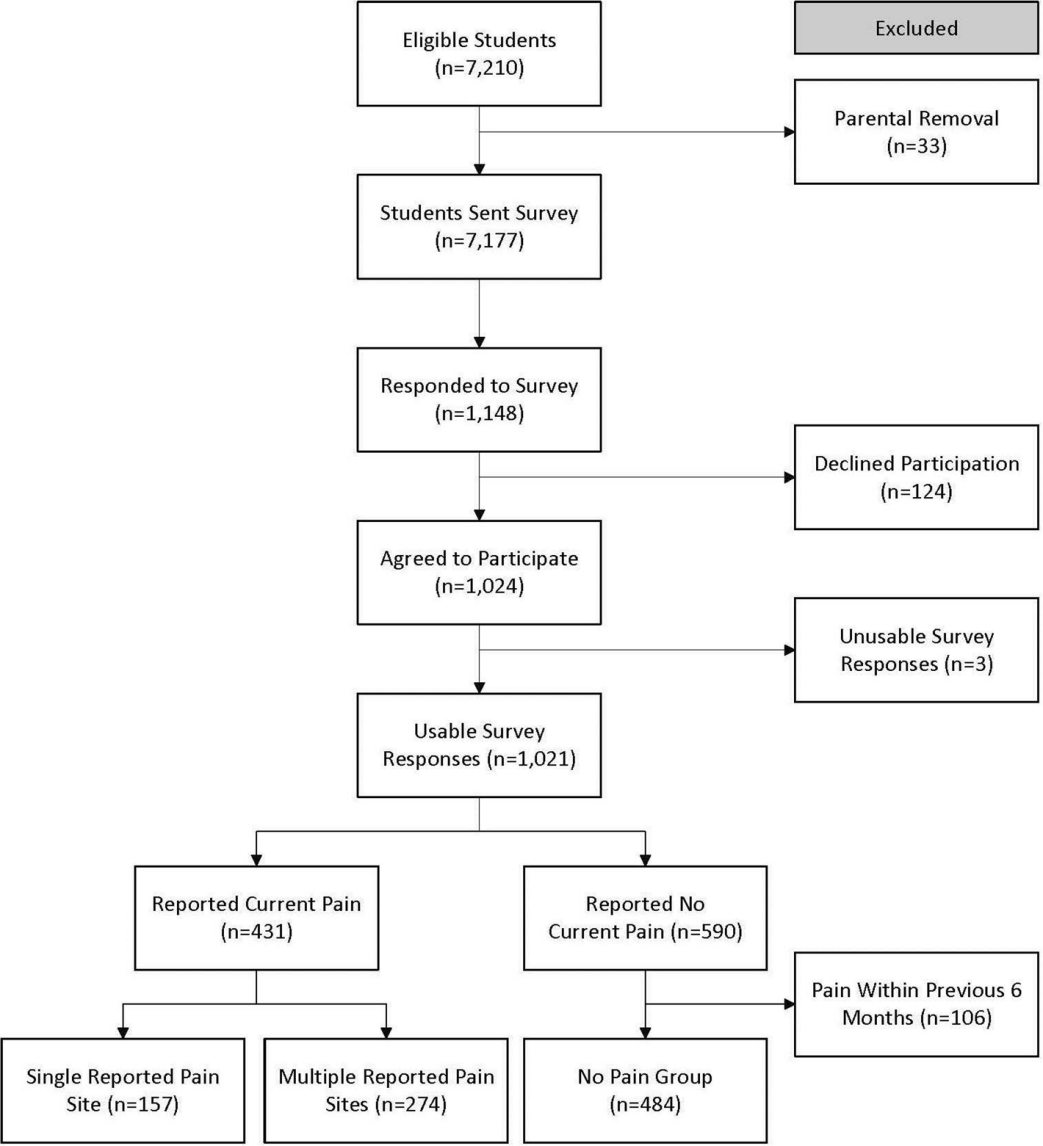
STROBE Flow Chart

A total of 1148 (16%) responded to the survey, with 1024 participants volunteering for the study (14.3%). Three participants’ data was unusable due to nonsensical responses and was removed. Of the 1021 participants, 431 (42.2%) reported current pain. Those who had no current pain but reported pain in the previous 6 months were excluded from this analysis (*n* = 106, 10.4%). Final group allocations (out of *n* = 915) were 484 (52.9%) controls, 157 (17.2%) with single-site pain, and 274 (29.9%) with multi-site pain (Fig. 1). The multi-site group had a median ± IQR of 3 ± 2 (range: 2–9) pain sites.

Our sample contained a significantly greater proportion of girls than boys in all pain groups (67.3% female, *p* < 0.001); however, pairwise comparisons did not demonstrate significant differences for sex between any of the groups. Groups were not significantly different in terms of mean age (*p* = 0.050), height (*p* = 0.686), weight (*p* = 0.783), or BMI (*p* = 0.702). Participant characteristics can be found in Table 1.

**Table 1:** Participant characteristics and comparisons among pain groups

	No Pain (*n* = 484)	Single Site Pain (*n* = 157)	Multi-Site Pain (*n* = 274)	Main Effect	NPvSSP	NPvMSP	SSPvMSP
Sex, N (%)	Girls: 302 (62.8%)	Girls: 111 (70.3%)	Girls: 216 (78.5%)	*p* < 0.001	ns	ns	ns
	Boys: 179 (37.2%)	Boys: 47 (29.7%)	Boys: 59 (21.5%)				
Age, (years)	14.6 ± 2.0 (14.5–14.8)	15.0 ± 1.9 (14.7–15.3)	14.9 ± 1.9 (14.7–15.2)	*p* = 0.050	na	na	na
Height (m)	1.65 ± 0.14 (1.63–1.66)	1.66 ± 0.11 (1.64–1.67)	1.65 ± 0.12 (1.64–1.66)	*p* = 0.686	na	na	na
Weight (kg)	61.2 ± 18.3 (59.6–62.9)	62.0 ± 16.0 (59.5–64.5)	62.1 ± 15.7 (60.2–64.0)	*p* = 0.783	na	na	na
BMI (kg/m2)	22.5 ± 5.6 (21.9–23.0)	22.7 ± 5.8 (21.8–23.7)	22.8 ± 5.2 (22.2–23.4)	*p* = 0.702	na	na	na

All variables reported as mean ± standard deviation (95% confidence interval), except for sex

*NP* No Pain, *SSP* Single-Site Pain, *MSP* Multi-Site Pain, *ns* not significant, *na* not performed

There was a significant difference between groups in health related quality of life (EQ-5D scores) (*p* < 0.001). The no pain group had significantly higher health related quality of life compared to those with single-site pain (mean difference 0.073 95% CI = 0.035–0.110, *p* < 0.001) and compared to the multi-site pain group (mean difference 0.166 95% CI = 0.135–0.197, *p* < 0.001). Those with multi-site pain has significantly lower quality of life compare to those with single site-pain (mean difference 0.094 95% CI = 0.052–0.134, p < 0.001). All quality of life data can be found in Table 2.

**Table 2:** Quality of life, sleep, and sports participation among pain groups

	No Pain (*n* = 484)	Single Site Pain (*n* = 157)	Multi-Site Pain (*n* = 274)	Main Effect	NPvSSP	NPvMSP	SSPvMSP
EQ 5D Index Score	0.834 ± 0.15 (0.820–0.847)	0.761 ± 0.17 (0.734–0.788)	0.667 ± 0.21 (0.642–0.693)	*p* < 0.001	*p* < 0.001	*p* < 0.001	*p* < 0.001
Sleep Hours	7.42 ± 1.4 (7.29–7.54)	7.07 ± 1.6 (6.82–7.31)	6.75 ± 1.6 (7.05–7.26)	*p* < 0.001	*p* = 0.041	*p* < 0.001	*p* = 0.099
Sports Participation, N Yes (%)	317 (65.9%)	118 (74.2%)	195 (70.9%)	*p* = 0.099	na	na	na
Sports Hours/Week	10.2 ± 6.9 (9.4–10.9)	11.2 ± 6.7 (10.0–12.5)	10.9 ± 8.8 (9.6–12.2)	*p* = 0.349	na	na	na

All variables reported as mean ± standard deviation (95% confidence interval), except for sports participation

*NP* No Pain, *SSP* Single-Site Pain, *MSP* Multi-Site Pain, *na* not performed

Sleep quantity was significantly different among groups (*p* < 0.001; Table 2), with pairwise comparisons showing significantly reduced sleep in those with single-site pain (mean difference = 0.35 h, 95% CI = 0.01–0.69, *p* = 0.041) and multi-site pain (mean difference = 0.67 h, 95% CI = 0.39–0.95, *p* < 0.001) compared to the no pain group. There was no difference between those with single- versus multi-site pain (mean difference = 0.32 h, 95% CI = -0.05–0.69, *p* = 0.099). There was a significant difference between groups in the proportion with sufficient, intermediate, or insufficient sleep (*p* < 0.001). Pairwise comparisons demonstrated that youth with multi-site pain had a significantly greater proportion of intermediate and insufficient sleep quality compared to no pain (*p* < 0.001 and *p* < 0.001, respectively) and single site pain (*p* = 0.004 and *p* = 0.004, respectively) groups. Sleep data is presented in Table 3.

**Table 3:** Sleep categories between pain groups

	No Pain (*n* = 484)	Single Site Pain (*n* = 157)	Multi-Site Pain (*n* = 274)	Main Effect	NPvSSP	NPvMSP	SSPvMSP
Sufficient	23 (4.9%)	3 (1.9%)	5 (1.8%)	*p* < 0.001	ns	ns	ns
Intermediate	275 (58.0%)	81 (51.9%)	98 (36.0%)		ns	*p* < 0.001	*p* = 0.004
Insufficient	176 (37.1%)	72 (46.2%)	169 (62.1%)		ns	*p* < 0.001	*p* = 0.004

*NP* No Pain, *SSP* Single-Site Pain, *MSP* Multi-Site Pain, *ns* not significant

No significant differences among the proportion of those participating in sports were found (*p* = 0.099). Significant differences among groups were also not found for sport participation hours per week (*p* = 0.349). All sport participation data can be found in Table 2.

**Fig. 2:**
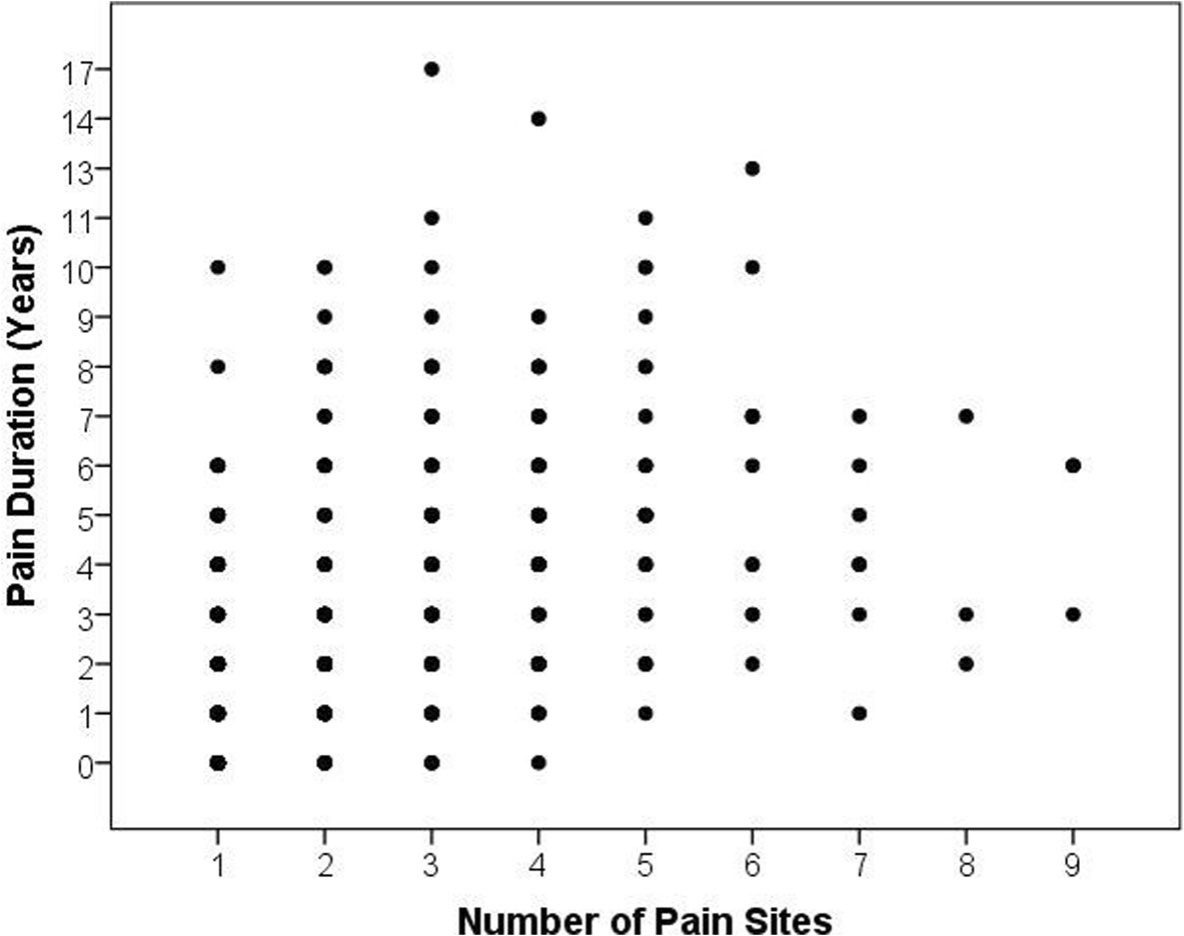
Scatterplot of pain duration relative to the number of pain sites. A positive, moderate, and significant correlation (*r* = 0.437, *p* < 0.001) was found

Average and worst pain (i.e. intensity) in the previous week was significantly (*p* = 0.005 and *p* = 0.005, respectively) greater in those with multi-site pain (4.34 ± 2.09 and 6.66 ± 2.29, respectively) compared to single site pain (3.76 ± 1.90 and 6.00 ± 2.16, respectively). The knee, lower back, and ankle were the most common pain sites for both single site pain and multi-site pain groups (Table 4). There were no significant differences (*p* = 0.065) in pain frequency between pain groups (Table 5). Duration of pain was also significantly greater (*p* < 0.001) in youth with multi-site pain (3.90 ± 2.75 years) compared to those with single site pain (1.78 ± 1.84 years). A positive, moderate, and significant correlation (*r* = 0.437, *p* < 0.001) was found between the pain duration and number of pain sites (Fig. 2).

**Table 4:** Pain location frequencies for pain groups, N(%)

	Single Site Pain	Multi-Site Pain
Knee	39 (24.8%)	157 (17.1%)
Lower Back	31 (19.7%)	149 (16.0%)
Ankle	28 (17.8%)	112 (12.2%)
Wrist/Hand	18 (11.5%)	90 (9.8%)
Neck	12 (7.6%)	102 (11.1%)
Shoulder	10 (6.4%)	93 (10.1%)
Hip	9 (5.7%)	92 (10.0%)
Upper Back	8 (5.1%)	106 (11.5%)
Elbow	2 (1.3%)	19 (2.1%)

**Table 5:** Frequency (%)of pain for pain groups

	Single Site Pain	Multi-Site Pain
Almost Daily	29.4%	42.0%
Several Times Per Week	25.5%	24.1%
Weekly	23.5%	15.3%
Monthly	13.1%	12.8%
Rarely	8.5%	5.8%

## Discussion

In this population based survey of adolescents, we found a high prevalence of pain, with knee pain being the most common. Those with MSK pain demonstrated poorer sleep quality and HRQoL, compared to those without pain. These assocoations were even stronger with those in pain in more than one location. Worryingly, a high proportion (nearly one third) reported pain in more than one location. This group of adolescents with multi-site pain may need particular focus, as in additon to poor sleep and HRQoL, they are characterized by high frequency and intensity of pain, compared to those with single-site pain. These factors have been indicated to increase the risk of a poor prognosis [5, 25].

This study confirms previous evidence indicating that knee pain is the single most common MSK pain location among adoelscents [11]. However the results indicate the commonality of multi-site in this population- nearly twice as many as those with pain in a single location. Perhaps unsurprisingly multisite pain was associated with a longer pain duration (despite no difference in age). This may suggest that having pain in one location is a risk factors for developing MSK pain in another location [26]. The high number of participants, and low HRQoL in this group underscores the need need for a paradigm shift away from looking at isolated pain complaints and to focus on how best to manage adolescents with pain in multiple locations.

Previously, in a population based sample of adolescents [27], those classified as having multi-site bodily pain (predominantly knee, back, head, stomach) are often females with a low HRQoL and lower sports participation than other pain patterns such as localized pain. These characteristics are similar in those with mulit-site pain in the current study. This may cause for concern, as having more multi-site and/or widespread pain [28–30], longer pain duration [28, 30], and pain intensity [29, 30] have all been associated with worse prognosis across a range of different MSK pain conditions, and in adolescents are associated with pain and functional limitations after 5 years study (Holden et al. in review, prog-paper). Further research should investigate if identifying these common profiles or characteristics early on can help identify the adolescents who are most in need of attention.

A high volume of sports participation has previously been linked to overuse pain and injury in youth [31, 32]. In general, injured youth completing more organized sports hours per week than those compared to those who were not injured [31]. The weekly hours of sports participation in our study (overall = 10.2 ± 6.9) are similar to levels previously reported (uninjured = 9.1 ± 6.3, injured = 11.2 ± 2.6) study [31]. However, we did not find differences in sports participation between groups. This difference could be attributed to the use of a diagnosed injury (both chronic and acute) vs self-reported pain (which can also be associated high sedentary time [33]) in our study. Future research should investigate the relationships among hours of sport participation, injury status, and chronic pain in this population, including non-linear relationships.

It has been proposed that adolescence is a critical developmental period in which small investments in health promotion, or ‘nudges’ in health behaviours can have impacts across the life-span [34, 35]. One critical health related phenomenon during this period is sleep. According to the National Sleep Foundation, adolescents need 8–9 h of sleep per night [36]. In our study, nearly 60% of the adolescents with multi-site pain reported insufficient sleep (6.75 h), which is a cause for concern as it is higher than 45% of the general youth population who get insufficient sleep, reported by the National Sleep Foundation [36]. Sleep and pain have a complex intertwining recipricol relationship- in the short term acute lack of sleep is associated with subsequent worse pain [37, 38], but in the longer term decreased sleep quantity and quality is an independant risk factors for both the onset and prognosis of pain [25, 39]. However, the lack of sleep these adolescents experience can go beyond pain and can have wider implications for health due to the association between sleep problems and psychological factors [40].

In a Danish sample, Rathleff et al. [11] found that single-site pain was twice as common as multi-site pain, but it is the reverse in this sample with multi-site being more common. The difference may be due to differences in the populations studied. Further due to the low response rate in the current study, it is unknown if this finding is as a result of response bias (i.e. those with more pain more likely to respond to questionnaire). Further the lower HRQoL in this study, with pain-free adolescents from the current study having similar values as those with pain from the Danish population. Overall, the HRQoL was low compared to other studies, also among those without pain. Perhaps this is linked to poor sleep, and associated psychological problems that may be expected, but this is speculative and future research is needed to understand this.

The response rate was low, but not deemed a major threat to the validity of our findings as this cross-sectional study aimed to compare pain characteristics (intensity, duration), quality of life, sleep, sport participation between adolescents those with single site pain, multi-site pain and those without pain, rather than estimate the prevalence of pain complaints. The difference in response rates between sexes seems consistent with females being more vigilant about pain and seeking medical care at a higher rate than males [41]. This study relied on self-reported data, similar to previous studies. This may cause unknown bias towards some the exposures we collected. However, as the adolescents where not aware of our main hypothesis, we expect this to equally affect all adolescents.

## Conclusions

A large percentage of youth (10–18 years) experience regular pain that greatly reduces their self-reported quality of life and sleep quantity and quality. Those reporting multi-site pain report a greater reduction in quality of life and increased pain intensity and duration. The duration of pain is related to the number of pain sites, possibly demonstrating a rational for earlier intervention in those with single-site pain. Complaints of pain in youth should be disregarded with caution.

## Data Availability

The datasets used and/or analysed during the current study are available from the corresponding author on reasonable request.

## Abbreviations

EQ-5D: EuroQol Group 5-Dimensional 3 levels Self-Report Questionnaire
HRQoL: Health-related quality of life
MSK: Musculoskeletal
